# Observations on the Therapeutic Use of Alcohol

**Published:** 1882-11

**Authors:** 


Some Observations on the Therapeutic Use of Alcohol. By Alfred K. Hills, M. D., New York. Reprinted from the *New York Medical Times.* 12mo. Pp. 27.

Dr. Hills gives an intelligent and useful discussion of the physiological and medicinal effects of alcohol. Such a paper is needed, for physicians woefully abuse alcoholic drinks. Many a typhoid patient has been sent to join "the great majority" from its abuse. A better acquaintance with its physiological effects would probably amend its wholesale exhibition. It is seldom or never needed.

